# 2-Phenylacetamide Isolated from the Seeds of *Lepidium apetalum* and Its Estrogen-Like Effects In Vitro and In Vivo

**DOI:** 10.3390/molecules23092293

**Published:** 2018-09-07

**Authors:** Mengnan Zeng, Meng Li, Miao Li, Beibei Zhang, Benke Li, Li Zhang, Weisheng Feng, Xiaoke Zheng

**Affiliations:** 1 Department of Medicine, Henan University of Chinese Medicine, Zhengzhou 450046, China; 17320138484@163.com (M.Z.); limeng31716@163.com (M.L.); limiao1206sunny@163.com (M.L.); zhangs9426@163.com (B.Z.); libk2017@163.com (B.L.); sonny.fairy.love@163.com (L.Z.); fwsh@hactcm.edu.cn (W.F.); 2 Collaborative Innovation Center for Respiratory Disease Diagnosis and Treatment & Chinese Medicine Development of Henan Province, Zhengzhou 450046, China

**Keywords:** *Lepidium apetalum* Willd, 2-phenylacetamide, estrogen-like effects, ER*α*, ER*β*, GPR30

## Abstract

The aim of this study was to investigate the estrogen-like effects of 2-phenylacetamide (PA), which is the main compound isolated from the seeds of *Lepidium apetalum* Willd (LA). Results showed that LA and PA could promote the proliferation of MCF-7 cells. The mouse uterine weight test showed that, LA and PA could increase the uterus index of immature female mice, and the levels of luteinizing hormone (LH) and estrogen (E2). LA could increase the expression of ER*α* and ER*β*, while PA could increase the expression of ER*α*, ER*β* and GPR30 in the uterus and MCF-7 cells. In addition, co-incubation of the estrogen receptor blocker with LA or PA abolished the inductive effect of the proliferation. PA has estrogenic activities and was the material basis of LA that played the estrogenic effect. LA and PA might be used for the treatment of perimenopause syndrome in a novel application.

## 1. Introduction

Estrogen is an important regulatory hormone in women’s bodies. It is a class of steroids, mainly produced by the ovary and placenta. After women enter menopause, estrogen levels rapidly decline, leading to postmenopausal osteoporosis and perimenopausal syndrome. Estrogen replacement therapy (ERT) is used for treatment in clinical studies [1]. However, the long-term use of this therapy has obvious side effects [2]. Therefore, the safer and more effective phytoestrogen (PE) has received increasing attention from researchers. In addition, estrogen receptors also mediate the effects of PE which include the genomic nuclear estrogen receptors (ER*α* and ER*β*) and the nongenomic estrogen receptor (GPR30) [3].

The dry mature seeds of *Lepidium apetalum* Willd (LA) which belong to the Brassicaceae family have been used to relieve coughs, prevent asthma, reduce edema and promote urination in traditional Chinese medicine (TCM). Oils, flavonoids, sterols and cardiac glycosides [4,5,6] are contained in the seeds of *L. apetalum* Willd. In addition, it shows anti-oxidant, antibacterial and cardiac activities [7]. Clinical research suggests that it can be used for heart failure [8,9], pigmentation [10] and oral cancer [11]. In our preliminary study, we found that several Chinese herbal medicines showed estrogen-like effects, including LA. Then, we isolated the main compounds from LA and obtained 2-phenylacetamide (PA) [12] and some other compounds and examined their estrogen-like activities. However, the estrogen-like effects of LA and PA have not yet been reported. Therefore, in this study, we evaluated the estrogen-like effects of LA and PA in vitro and in vivo, and explored the potential mechanisms.

## 2. Results

### 2.1. Effect of LA on MCF-7 Cell Proliferation

**Figure 1:**
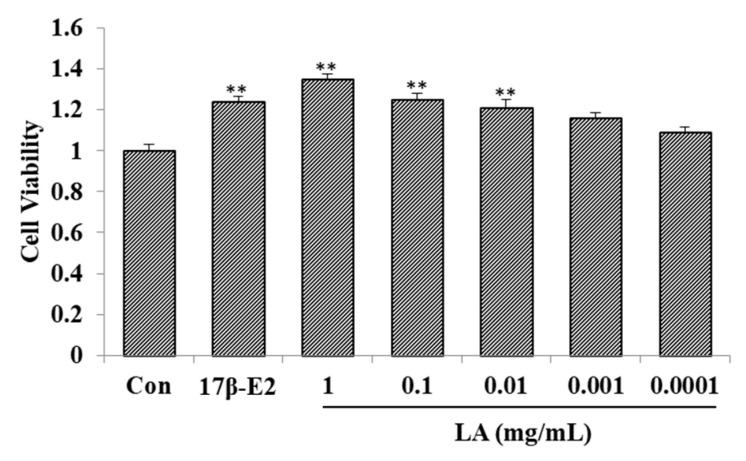
Effect of *Lepidium apetalum* Willd (LA) on MCF-7 cell proliferation (*x* ± SD, *n* = 3). * *p* < 0.05; ** *p* < 0.01 compared to controls.

As shown in Figure 1, compared with the control group, 1–0.01 mg/mL of LA improved the proliferation rate of the MCF-7 cell line. In addition, 17*β*-estradiol (17*β*-E2; 1 μM) improved the proliferation rate of the MCF-7 cell line.

### 2.2. Effects of LA on Immature Female Swiss Mice

As shown in Table 1, EV (estradiol valerate, the positive drug of the animal experiment) significantly increased the uterus coefficient in mice, as well as the levels of LH, follicle stimulating hormone (FSH) and E2. Compared with the control group, LA-L (446 mg/kg) and LA-H (892 mg/kg) significantly increased the uterus coefficient. LA-L and LA-H significantly increased the levels of E2 and LH.

### 2.3. Effects of LA on the Expression of Estrogen Receptors

**Figure 2:**
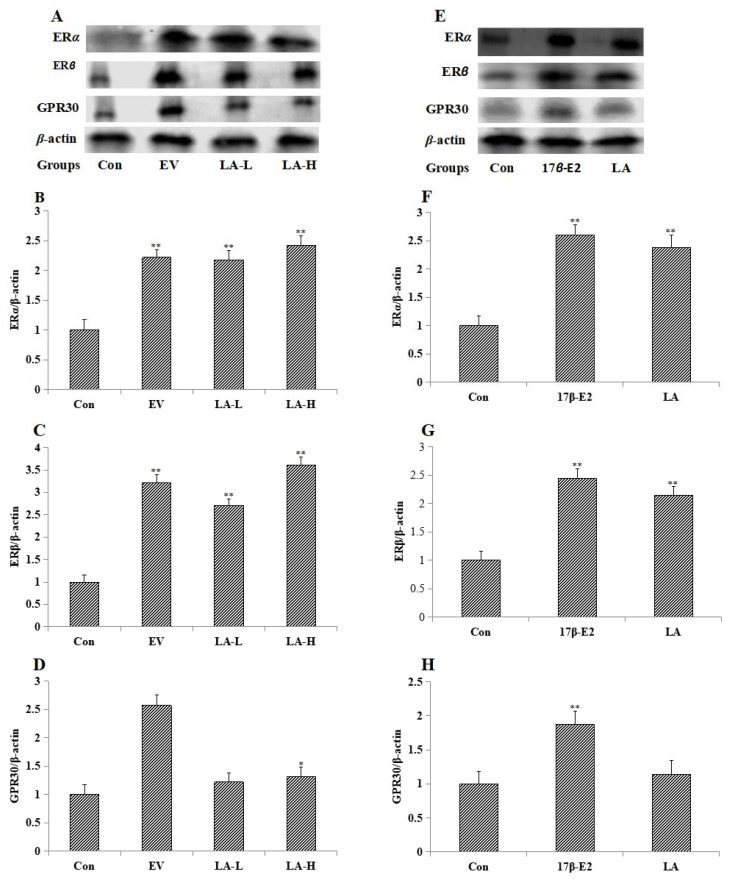
Western blot analysis of the expression of ER*α*, ER*β* and GPR30 in the uterus of immature female Swiss mice and MCF-7 cells (*n* = 3). Figure (**A**), (**B**), (**C**) and (**D**) show the expression of ER*α*, ER*β* and GPR30 in the uterus. Figure (**E**), (**F**), (**G**) and (**H**) show the expression of ER*α*, ER*β* and GPR30 in MCF-7 cells. * *p* < 0.05; ** *p* < 0.01 compared to controls.

LA-L (446 mg/kg) and LA-H (892 mg/kg) enhanced the expression of estrogen nuclear receptors (ER*α* and ER*β*) in the uterus (Figure 2A–D). LA (10^−2^ mg/mL) enhanced the expression of estrogen nuclear receptors in MCF-7 cells (Figure 2E–H). EV (0.33 mg/kg) and 17*β*-E2 (10^−6^ mol/L) enhanced the expression of estrogen nuclear receptors and GPR30 in uterus and MCF-7 cells separately.

### 2.4. Effect of Estrogen Receptor Antagonists on LA Aroused MCF-7 Cell Proliferation

**Figure 3:**
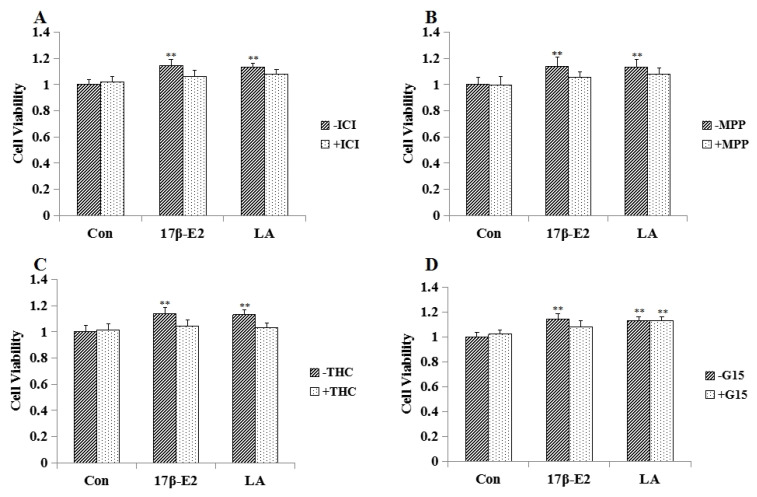
Effect of estrogen receptor antagonists on LA aroused MCF-7 cell proliferation (*n* = 3). (**A**) Effects of unspecific ER antagonist (ICI182780) on LA stimulated MCF-7 cell proliferation. (**B**) Effects of methylpiperidino-pyrazole (MPP) on LA stimulated MCF-7 cell proliferation. (**C**) Effects of Delta (9)-tetrahydrocannabinol (THC) on LA stimulated MCF-7 cell proliferation. (**D**) Effects of specific GPR30 antagonist G-15 (G-15) on LA stimulated MCF-7 cell proliferation. * *p* < 0.05; ** *p* < 0.01 compared to controls.

As shown in Figure 3, ICI 182780 (unspecific ER antagonist, 1 μM), methylpiperidino-pyrazole (MPP; specific ER*α* antagonist; 1 μM), Delta (9)-tetrahydrocannabinol (THC; specific ER*β* antagonist; 1 μM) could block the effect of LA (10^−2^ mg/mL) on MCF-7 cell proliferation. ICI 182780 (1 μM), MPP (1 μM), THC (1 μM) and G-15 (specific GPR30 antagonist G-15, 1 μM) could block the effect of 17*β*-E2 on MCF-7 cells.

### 2.5. Effect of PA on MCF-7 Cell Proliferation

**Figure 4:**
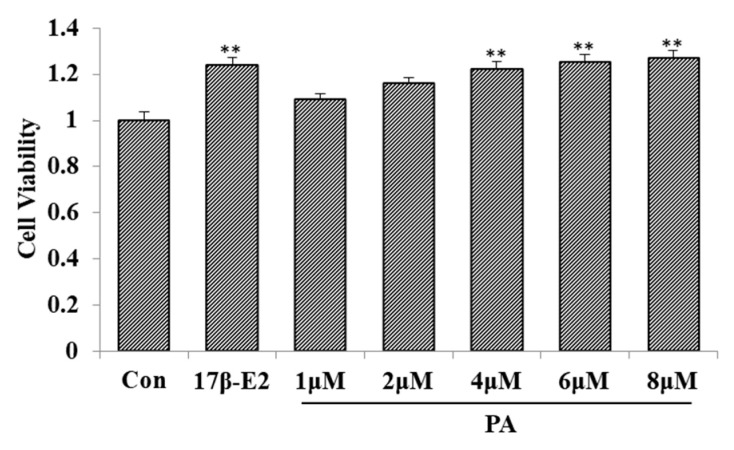
Effect of 2-phenylacetamide (PA) on MCF-7 cell proliferation (*x* ± SD, *n* = 3). ** *p* < 0.01 compared to control using a Student’s *t*-test.

Figure 4 shows that 4–8 μM of PA improved the proliferation rate of the MCF-7 cell line.

### 2.6. Effect of PA on MCF-7 Cell Proliferation

Table 2 shows that EV and LA significantly promoted the uterus coefficient, LH and E2. Compared with the control group, PA-L and PA-H significantly promoted the uterus coefficient, LH and E2.

### 2.7. The Effect of PA on MCF-7 Cell Proliferation

**Figure 5:**
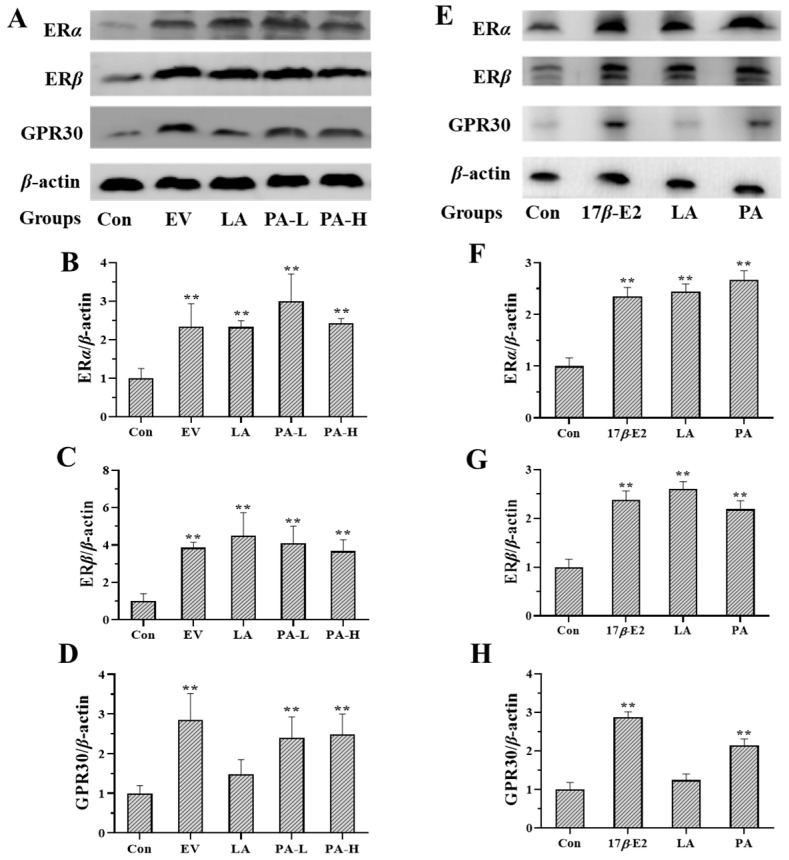
Western blot analysis of the expression of estrogen receptors in the uterus of immature female Swiss mice and MCF-7 cells (*n* = 3). Figure (**A**–**D**) show the expression of ER*α*, ER*β* and GPR30 in the uterus. Figure (**E**–**H**) show the expression of ER*α*, ER*β* and GPR30 in MCF-7 cells. ** *p* < 0.01 compared to controls.

PA-L (25 mg/kg) and PA-H (50 mg/kg) enhanced the expression of estrogen nuclear receptors and GPR30 in uterus (Figure 5A–D). PA (5 μM) upgraded the expression of estrogen nuclear receptors and GPR30 in MCF-7 cells (Figure 5E–H).

### 2.8. Effect of Estrogen Receptor Antagonists on PA Aroused MCF-7 Cell Proliferation

**Figure 6:**
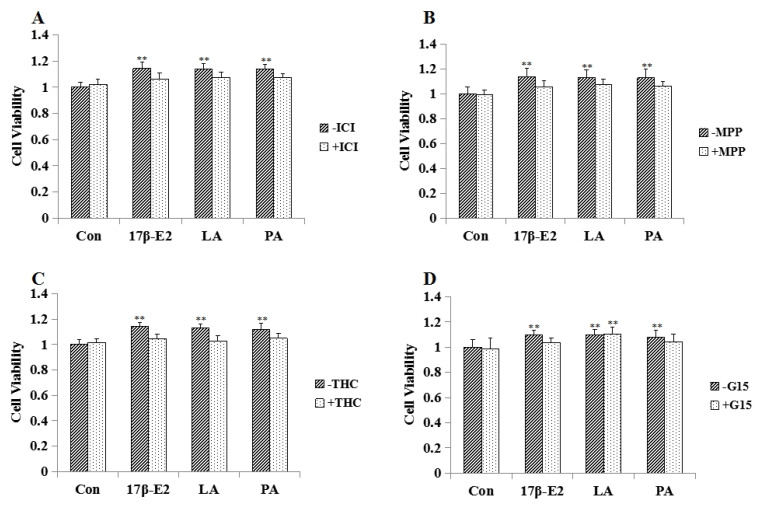
Effect of estrogen receptor antagonists on PA aroused MCF-7 cell proliferation (*n* = 3). (**A**) Effects of unspecific ER antagonist (ICI182780) on PA stimulated MCF-7 cell proliferation. (**B**) Effects of methylpiperidino-pyrazole (MPP) on PA stimulated MCF-7 cell proliferation. (**C**) Effects of Delta (9)-tetrahydrocannabinol (THC) on PA stimulated MCF-7 cell proliferation. (**D**) Effects of specific GPR30 antagonist G-15 (G-15) on PA stimulated MCF-7 cell proliferation. * *p* < 0.05; ** *p* < 0.01 compared to controls.

As shown in Figure 6, ICI 182780 (unspecific ER antagonist, 1 μM), MPP (specific ER*α* antagonist, 1 μM), THC (specific ER*β* antagonist, 1 μM) and G-15 (specific GPR30 antagonist G-15, 1 μM) could block the effect of PA (5 μM) on MCF-7 cell proliferation.

## 3. Discussion

Irregular menstruation and amenorrhea may occur when estrogen levels have decreased. After entering menopause [13], there may be a series of diseases and symptoms, such as menstrual disorders, mood swings, hot flushes and cardiovascular disease. Estrogen replacement therapy is used for treatment in clinical studies [14]. However, the long-term use of this therapy has obvious side effects [15]. Therefore, safer and more effective phytoestrogen has received increasing attention from researchers. Phytoestrogens play a two-way role in changing and promoting estrogen activity. When the levels of estrogen are low in vivo, phytoestrogens have an estrogen-like effect; when the levels are high, they exert antiestrogenic activity by competitively binding to estrogen receptors [16,17]. Therefore, phytoestrogens are known as natural, selective estrogen receptor modulators. Nourishing plants and seed medicines are normally used as the phytoestrogens in traditional Chinese medicine. In a previous study from our laboratory, it was found that the water extract of LA, a kind of seed medicine, had an estrogen-like effect. In the following experiment, we investigated the estrogen-like effects of compounds isolated from LA, and found that PA, which is one of the main compounds of LA, was the material basis of LA that played the estrogenic effect. In addition, PA is the key pharmaceutical intermediate of atenolol [18] and penicillin [19].

Uterine coefficient of immature rabbits was used to detect estrogenic activity of drugs in some of the earliest studies [20,21]. This was until Astwood and others [22,23] determined that the uterine coefficient of immature mice is more sensitive than that of immature rabbits. Uterine coefficient (the ratio of weight of the uterus to body weight) in immature mice was used to evaluate estrogen-like activity in our study. LA and PA could significantly increase the uterus coefficient, LH and E2, indicating that the LA and PA had an estrogen-like effect.

MCF-7 cells are the most commonly used cell lines for detecting estrogen-like activity. Estrogen-like substances can promote the proliferation of MCF-7 cells which are estrogen receptor-positive cells [24]. In our study, LA and PA promoted the proliferation of MCF-7 cells and exhibited dose-dependent behavior. In another experiment, estrogen receptor antagonists (ICI182, 780, MPP, THC and G-15) [25,26,27,28] were used to investigate the mechanisms of LA and PA. Estrogen nuclear receptor antagonists could block the effect of LA on MCF-7 cells, demonstrating that the estrogen-like activity of LA was mediated by estrogen nuclear receptors (ER*α* and ER*β*). Estrogen nuclear receptor antagonists and G-15 could block the effect of PA on MCF-7 cells indicating the estrogen-like activity of PA was mediated by estrogen nuclear receptors and GPR30.

Furthermore, Western blot showed that PA (5 μM) promoted the expression of ER*α*, ER*β* and GPR30 in MCF-7 cells, and that PA (25, 50 mg/kg) promoted the expression of ER*α*, ER*β* and GPR30 in uterine. LA (10^−2^ mg/mL) promoted the expression of ER*α* and ER*β* in MCF-7 cells, LA (446, 892 mg/kg) promoted the expression of ER*α* and ER*β* in uterus. This indicates that PA might exert an estrogen-like effect through estrogen nuclear receptors and GPR30, while LA might exert an estrogen-like effect through estrogen nuclear receptors, EV (0.33 mg/kg) and 17*β*-E2 (1 μM) just as the positive control.

In addition, uterine edema and hyperplasia was found in the EV group which may be because estrogen can promote the expression of vascular endothelial growth factor (VEGF) in the uterus, enhance blood vessel permeability, induce overgrowth of endothelial cells and inhibit endothelial cells apoptosis [29]. In our study, LA and PA did not have the adverse reactions of EV.

In conclusion, our studies in vitro and in vivo all proved that LA has an estrogen-like effect, and PA, which is one of the main compounds of LA, was the material basis of LA that played the estrogenic effect. ICI182780, MPP, THC and G-15 estrogen receptor antagonist experiments, together with Western blot, revealed that the estrogen-like effects of LA are mediated by estrogen nuclear receptors, and the estrogen-like effects of PA are mediated by estrogen nuclear receptors and GPR30. In addition, LA and PA had no artificial side effects compared to synthetic estrogen. LA and PA may be used for the treatment of perimenopause syndrome in a novel application, and is easy to obtain and synthesize, making it suitable for industrial production.

## 4. Materials and Methods

### 4.1. Plant Material

**Figure 7:**
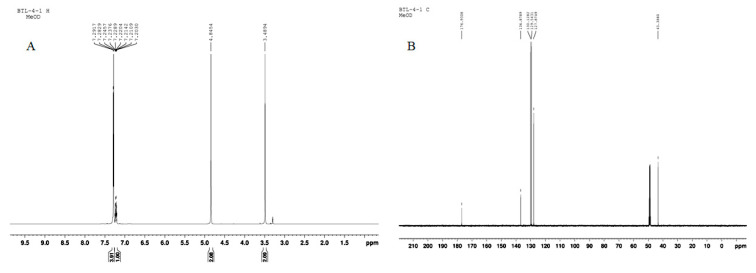
The ^1^H-NMR (**A**) and ^13^C-NMR (**B**) spectrums of PA (in CD_3_OD). The data of NMR are as follows: ^1^H-NMR (CD_3_OD, 500 MHz) *δ*:7.29 (5H, m, H-2,3,4,5,6), 3.50 (2H, s, H-7); ^13^C-NMR (CD_3_OD, 125 MHz) *δ*:136.9 (C-1), 130.1 (C-2,6), 129.6 (C-3,5), 127.9 (C-4), 43.4 (C-7), 177.0 (C-8).

**Figure 8:**
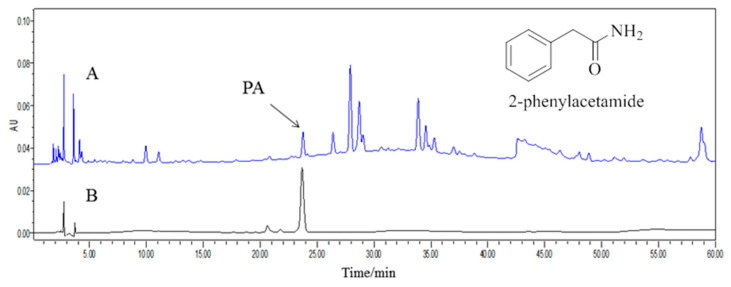
Representative HPLC chromatogram of PA (**A**) and crude water extract of LA (**B**). Waters Alliance 2695 separations module equipped with Empower software hyphened with quaternary pumps, an automatic injector, a Waters 2998 photodiode array (PDA) detector at 190–800 nm, and a 250 mm × 4.6 mm × 5 μm Platisil ODS C18 column was used for the separation. HPLC analysis conditions: Acetic acid water ((A), 0.1%)-acetonitrile (B), 0~60 min (5~25% (A)); flow velocity: 1 mL/min, 5 μL injection; wavelength of detection: 254 nm; column temperature: 30 °C.

LA was collected from Nanyang City, China, and identified by Professor Dong Chengming, Henan University of Chinese Medicine. It (No. 20150715A) was deposited in the Science Experiment Center, Henan University of Chinese Medicine. The processed LA (8.0 kg) was extracted three times with H_2_O (80 L × 3, 1.5 h each time) at 100 °C. Evaporation of the solvent under reduced pressure yielded aqueous extracts (1.04 kg), which were then precipitated at an ethanol concentration of 80%. The liquid supernatant was concentrated in a vacuum evaporator to yield gross extract (628 g), which was suspended in H_2_O (1.5 L). The water-soluble substances were subjected to a Diaion HP-20 macroporous resin column and eluted with EtOH:H_2_O (0:100, 20:80, 40:60) successively to obtain three fractions (F1–F3). F3 (89.6 g) was suspended in H_2_O, followed by the removal of insoluble materials by centrifugation. After concentration, the water-soluble substances were (approximately 35 mL) subjected to Toyopearl HW-40 column chromatography elution with H_2_O and 10% MeOH-H_2_O to produce fractions (F3.1–F3.2). F3.2 (10.3 g) was dissolved in MeOH and after standing for 24 h, colorless crystals (4.3 g) were obtained. The purity of the colorless crystals was determined to be above 98% by HPLC. The structure was determined on the basis of NMR spectra and characterized as PA (Figure 7). The purity was estimated directly from the NMR spectrum and HPLC analysis to be greater than 99%. PA was verified as a genuine substance since it was detectable by assays of HPLC in the LA water extract prior to the separation procedure (Figure 8).

### 4.2. The Effects of LA and PA on MCF-7 Cell Proliferation

MCF-7 cells were cultured in hormone-free medium (HyClone, Logan, UT, USA) with 10% (V/V) charcoal-stripped fetal bovine serum (HyClone, Logan, UT, USA). There were 5000 cells per well in 96 well plates. Cells were separately exposed to 17*β*-Estradiol (17*β*-E2; Sigma, Louis, MO, USA; 1 μM), LA (1, 0.1, 0.001 and 0.0001 mg/mL) and PA (1, 2, 4, 6 and 8 μM) for 24 h. Cell viability was detected by 3-(4,5-dimethyl-2-thiazolyl)-2,5-diphenyl-2-H-tetrazolium bromide (MTT; Sigma, Louis, MO, USA).

### 4.3. The Effects of ICI182780, MPP, THC and G15 on LA and PA Promoted MCF-7 Cell Proliferation

The ER-unspecific antagonist ICI182780 (Tocris, Bristol, UK; 1 μM), the specific ER*α* antagonist methylpiperidino-pyrazole (MPP; Tocris, Bristol, UK; 1 μM), the specific ER*β* antagonist Delta (9)-tetrahydrocannabinol (THC; Tocris, Bristol, UK; 1 μM) and the seven-transmembrane domain protein G protein-coupled receptor (G15; Tocris, Bristol, UK; 1 μM) were added 30 min before treatment of 17*β*-E2, LA or PA. Other experimental steps are as described above in Section 4.2.

### 4.4. Animals

The study was conducted in accordance with the Regulations of Experimental Animal Administration issued by the State Committee of Science and Technology of the People’s Republic of China. Beijing Vital River Laboratory Animal Technology Co., Ltd. (Ethical approval reference number: SCXK2016-0011) provided us with immature female Swiss mice (4 weeks). All mice were housed in cages on a 12:12 h light-dark schedule at a controlled temperature (22 °C) with free access to food and water at the Laboratory Animal Research Center of Henan University of Chinese medicine. All the procedures for the care of the mice were in accordance with the institutional guidelines for animal use in research. A total of 40 mice (9–11 g) were divided into 4 groups: Control group, Estradiol Valerate group (EV; Bayer medical, Shanghai, China; 0.33 mg/kg), low dose of LA group (LA-L, 446 mg/kg), and high dose of LA group (LA-H, 892 mg/kg), continuous gavage for 7 d. Blood was collected by heart punctures. The uterus was peeled off quickly and weighed on a one millionth balance. The mouse uterine weight gain test of PA was the same as LA.

### 4.5. Western Blot

Proteins of uterine and MCF-7 cells were extracted using a mammalian protein extraction kit (Beijing ComWin Biotech Co., Ltd., Beijing, China) and quantified using the Bradford protein assay kit (Wuhan Boster Biological Technology, Ltd., Wuhan, China). Protein samples separated by SDS-PAGE were transferred to a PVDF membrane. The PVDF membrane was incubated with a primary antibody [ERα (Ac026; ABclnoal, Wuhan, China) 1:500; ER*β* (A2546; ABclnoal, Wuhan, China) 1:500; GPR30 (A10217; ABclnoal, Wuhan, China) 1:500] overnight at 4 °C, incubated with a secondary antibody (1:1000) for 1 h at 25 °C. We collected protein bands with chemiluminescence apparatus (Azure c500), and analyzed bands with Quantity One software.

### 4.6. Enzyme-Linked Immuno Sorbent Assay

The levels of E2, FSH and LH in serum were detected by ELISA (R&D, Minneapolis, MN, USA) according to manufacturer’s instructions.

### 4.7. Statistical Analysis

The results were expressed as the mean ± SD. The statistical differences between the control and the test fractions were assessed by analysis of variance (ANOVA) followed by a Student’s *t*-test for multiple comparisons. The obtained data were compared with the negative control group and values of *p* < 0.05 were considered significant.

## 5. Conclusions

LA had an estrogen-like effect, and PA, which is one of the main compounds of LA, was the material basis of LA that played the estrogenic effect. Estrogen-like effects of LA are mainly mediated by estrogen nuclear receptors, and the estrogen-like effects of PA are mainly mediated by estrogen nuclear receptors and GPR30. Further studies on model rats (animals with induced menopause/perimenopause syndrome/osteoporosis), including clinical trials, will have to show whether LA or its constituent, PA, have a potential for clinical treatment of postmenopause syndrome.

## Figures and Tables

**Table 1:** Effect of LA on immature female Swiss mice (*x* ± SD, *n* = 8).

Groups	Dose (mg/kg)	Uterus Coefficient (%)	LH (mIU/mL)	FSH (mIU/mL)	E2 (pmol/mL)
Con	—	0.1019 ± 0.015	3.68 ± 0.69	40.11 ± 4.81	32.82 ± 2.53
EV	0.33	0.2795 ± 0.027 **	4.77 ± 0.74 **	46.91 ± 2.37 **	39.32 ± 2.10 **
LA-L	446	0.1262 ± 0.021 *	5.22 ± 0.35 **	43.24 ± 4.36	45.06 ± 6.65 **
LA-H	892	0.1259 ± 0.022 *	5.42 ± 0.44 **	43.04 ± 5.26	46.11 ± 5.78 **

* *p* < 0.05; ** *p* < 0.01 compared to control using a Student’s *t*-test.

**Table 2:** Effect of PA on immature female Swiss mice (*x* ± SD, *n* = 8).

Groups	Dose (mg/kg)	Uterus Coefficient (%)	LH (mIU/mL)	FSH (mIU/mL)	E2 (pmol/mL)
Con	—	0.1009 ± 0.014	4.01 ± 0.58	39.77 ± 5.12	33.01 ± 2.14
EV	0.33	0.2395 ± 0.027 **	5.11 ± 0.49 **	46.91 ± 3.27 **	38.41 ± 2.07 **
LA	446	0.1253 ± 0.021 *	5.09 ± 0.61 **	42.37 ± 4.12	44.36 ± 4.37 **
PA-L	25	0.1219 ± 0.021 *	4.98 ± 0.47 **	42.34 ± 5.26	41.19 ± 5.57 **
PA-H	50	0.1201 ± 0.019 *	5.02 ± 0.79 **	41.14 ± 6.78	40.56 ± 6.01 **

* *p* < 0.05; ** *p* < 0.01 compared to control using a Student’s *t*-test.

## Abbreviations

PA	Phenylacetamide
LA	*Lepidium apetalum* Willd
ERT	Estrogen replacement therapy
TCM	Traditional Chinese medicine
PE	Phytoestrogen
MCF-7 cell	Breast adenocarcinoma cell line
LH	Luteinizing hormone
FSH	Follicle stimulating hormone
ICI182780	Faslodex
MPP	Specific ER*α* antagonist methylpiperidino-pyrazole
THC	Specific ER*β* antagonist Delta (9)-tetrahydrocannabinol
G15	Specific GPR30 antagonist
EV	Estradiol valerate
TBST	Tris buffered saline with Tween-20
MTT	Methyl thiazolyl tetrazolium
PBS	Phosphate buffer saline
DMSO	Dimethyl sulfoxide
17*β*-E2	17-beta-estradiol
PVDF	Poly vinylidene fluoride
VEGF	Vascular endothelial growth factor
