# Correction: Zeng et al. 2-Phenylacetamide Isolated from the Seeds of *Lepidium apetalum* and Its Estrogen-Like Effects In Vitro and In Vivo. *Molecules* 2018, *23*, 2293

**DOI:** 10.3390/molecules30224347

**Published:** 2025-11-10

**Authors:** Mengnan Zeng, Meng Li, Miao Li, Beibei Zhang, Benke Li, Li Zhang, Weisheng Feng, Xiaoke Zheng

**Affiliations:** 1 Department of Medicine, Henan University of Chinese Medicine, Zhengzhou 450046, China; 2 Collaborative Innovation Center for Respiratory Disease Diagnosis and Treatment & Chinese Medicine Development of Henan Province, Zhengzhou 450046, China

## Error in Figure

In the original publication [1], there was a mistake in Figure 5 as published. The bonds of ER*ɑ*, ER*β* and GPR30 were copied by mistake unintentionally and the corrected Figure 5A–D appears below. All authors have read and agreed to the published version of the manuscript. This correction was approved by the Academic Editor. The original publication has also been updated.

## Figures and Tables

**Figure 5:**
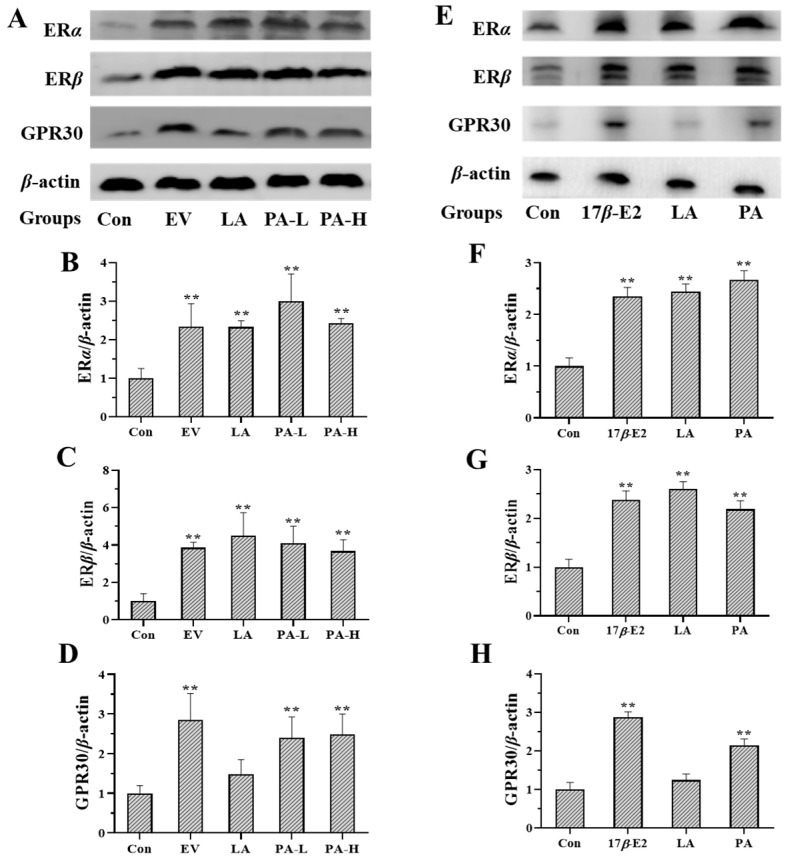
Western blot analysis of the expression of estrogen receptors in the uterus of immature female Swiss mice and MCF-7 cells (*n* = 3). Figure (**A**–**D**) show the expression of ER*α*, ER*β* and GPR30 in the uterus. Figure (**E**–**H**) show the expression of ER*α*, ER*β* and GPR30 in MCF-7 cells. ** *p* < 0.01 compared to controls.
